# High-fidelity SaCas9 identified by directional screening in human cells

**DOI:** 10.1371/journal.pbio.3000747

**Published:** 2020-07-09

**Authors:** Haihua Xie, Xianglian Ge, Fayu Yang, Bang Wang, Shuang Li, Jinzhi Duan, Xiujuan Lv, Congsheng Cheng, Zongming Song, Changbao Liu, Junzhao Zhao, Yu Zhang, Jinyu Wu, Caixia Gao, Jinwei Zhang, Feng Gu

**Affiliations:** 1 School of Ophthalmology and Optometry, Eye Hospital, Wenzhou Medical University, State Key Laboratory and Key Laboratory of Vision Science, Ministry of Health and Zhejiang Provincial Key Laboratory of Ophthalmology and Optometry, Wenzhou, Zhejiang, China; 2 Laboratory of Molecular Biology, National Institute of Diabetes and Digestive and Kidney Diseases, National Institutes of Health, Bethesda, Maryland, United States of America; 3 National Institute of Biological Sciences, Beijing, China; 4 The Second Affiliated Hospital and Yuying Children’s Hospital of Wenzhou Medical University, Wenzhou, Zhejiang, China; 5 Raymond G. Perelman Center for Cellular and Molecular Therapeutics, Children’s Hospital of Philadelphia, Philadelphia, Pennsylvania, United States of America; 6 Institute of Genomic Medicine, Wenzhou Medical University, Wenzhou, Zhejiang, China; 7 State Key Laboratory of Plant Cell and Chromosome Engineering, and Center for Genome Editing, Institute of Genetics and Developmental Biology, Chinese Academy of Sciences, Beijing, China; IMBA, AUSTRIA

## Abstract

CRISPR-*Staphylococcus aureus* Cas9 (CRISPR-SaCas9) has been harnessed as an effective in vivo genome-editing tool to manipulate genomes. However, off-target effects remain a major bottleneck that precludes safe and reliable applications in genome editing. Here, we characterize the off-target effects of wild-type (WT) SaCas9 at single-nucleotide (single-nt) resolution and describe a directional screening system to identify novel SaCas9 variants with desired properties in human cells. Using this system, we identified enhanced-fidelity SaCas9 (efSaCas9) (variant Mut268 harboring the single mutation of N260D), which could effectively distinguish and reject single base-pair mismatches. We demonstrate dramatically reduced off-target effects (approximately 2- to 93-fold improvements) of Mut268 compared to WT using targeted deep-sequencing analyses. To understand the structural origin of the fidelity enhancement, we find that N260, located in the REC3 domain, orchestrates an extensive network of contacts between REC3 and the guide RNA-DNA heteroduplex. efSaCas9 can be broadly used in genome-editing applications that require high fidelity. Furthermore, this study provides a general strategy to rapidly evolve other desired CRISPR-Cas9 traits besides enhanced fidelity, to expand the utility of the CRISPR toolkit.

## Abstract 2

CRISPR-SaCas9 technology offers promising solutions to treat genetic disorders by correcting pathogenic mutations, but off-target effects remain a major issue. This study characterizes the off-target activity of wild-type SaCas9 and uses a human cell-based screening system to identify high-fidelity SaCas9 variants, showing that they can dramatically reduce off-target effects.

## Introduction

The CRISPR-Cas9 system is a powerful tool for genome editing [1–3]. Among a wide array of potential applications, CRISPR-Cas9-mediated in vivo editing of human genome to correct disease-causing mutations is a promising approach for the treatment of numerous genetic disorders [4–6]. However, the off-target issue is a major challenge for reliable genome editing [7–9]. Specifically, the nuclease activity of Cas9 can be triggered by guide RNA targeting imperfectly matched, off-target genomic sites. This problem is particularly severe when the mismatches are located distal to the protospacer adjacent motif (PAM) sequence, a short stretch of nucleotides required for target selection [10–12]. These off-target effects not only confound interpretation of experimental results in the laboratory but also severely undermine the safety and reliability of clinical applications of the technology, where introduction of undesired mutations can lead to significant complications. Such off-target effects could also confound the usage of base editors [13], a newly developed genome-editing technology based on a catalytically impaired Cas9 and deaminase [14,15].

To address this key issue, various strategies have been adopted to reduce off-target effects of the most commonly used *Streptococcus pyogenes* Cas9 (SpCas9) nuclease. These include using Cas9 nickase mutants to create a pair of juxtaposed single-stranded DNA nicks [16], using a pair of catalytically inactive Cas9 nucleases, each fused to a FokI nuclease domain [17], delivering Cas9 as ribonucleoprotein (RNP) complexes [18], and truncating the guide sequence at the 5′ end [19]. Some groups reported higher-fidelity CRISPR-SpCas9 variants through structure-guided rational design [20–24] or random mutagenesis in yeast [25]. However, these efforts have largely been limited to SpCas9, which is too large for effective delivery into cells using adeno-associated virus (AAV).

AAV vectors are attractive gene-delivery vehicles due to their low immunogenic potential, reduced oncogenic risk from host-genome integration, and broad range of serotype compatibility [26]. However, the relatively small cargo size (about 4.5 kb) of AAV restricts the packaging of the commonly used SpCas9. In order to miniaturize Cas9 to facilitate its cellular delivery by AAV, SaCas9 was identified and further developed for in vivo genome editing [5]. Furthermore, our previous study suggested that SaCas9 possesses higher cleavage activity than SpCas9 in human cells [27], indicating the additional advantage of SaCas9 for in vivo genome editing.

The crystal structure of CRISPR-SaCas9 has been determined [28]. However, so far, high-fidelity variants of SaCas9 directly identified from human cells have not been reported. To create readily deliverable, high-fidelity SaCas9 (SaCas9-HF) variants, we first generated a SaCas9 variant library and developed a generally applicable directional screening system to isolate novel SaCas9 variants with reduced off-target effects in human cells. Using this method, we identified and validated a novel SaCas9 variant (Mut268) which possesses significantly higher fidelity without compromising its cleavage activity. Structural analysis suggested that Mut268 appears to loosen SaCas9’s grip on the guide RNA-DNA heteroduplex thus reducing the chance of off-target cleavage.

## Results

### Delineation of off-target effect of wild-type SaCas9 at the single-nucleotide level

Before searching for SaCas9-HF, we took advantage of our previously reported system based on CRISPR-Cas9-mediated *EGFP* inactivation to delineate the off-target effect of wild-type (WT) SaCas9 at the single-nucleotide level [29–32]. In this system, we could efficiently induce insertion or deletion mutations (in/dels) in an *EGFP* reporter gene and also easily detect CRISPR-SaCas9 activity targeting *EGFP* sequence based on flow cytometry (FCM). We optimized the plasmid amount for transfection and found 750 ng of all-in-one plasmids (pX601) is suitable (S1A Fig). We selected 5 different sites throughout the *EGFP* gene and designed perfectly matched guide sequences to test the performance of CRISPR-SaCas9 (S1 Table). The average percentages of *EGFP* disruption for the 5 sites were 49.3%, 38.2%, 53.5%, 45.8%, and 21.2% for site 1 through site 5, respectively (S1B Fig). Thus, we selected the first 4 sites with higher cleavage efficiencies to further characterize the DNA-targeting specificity of SaCas9.

**Fig 1:**
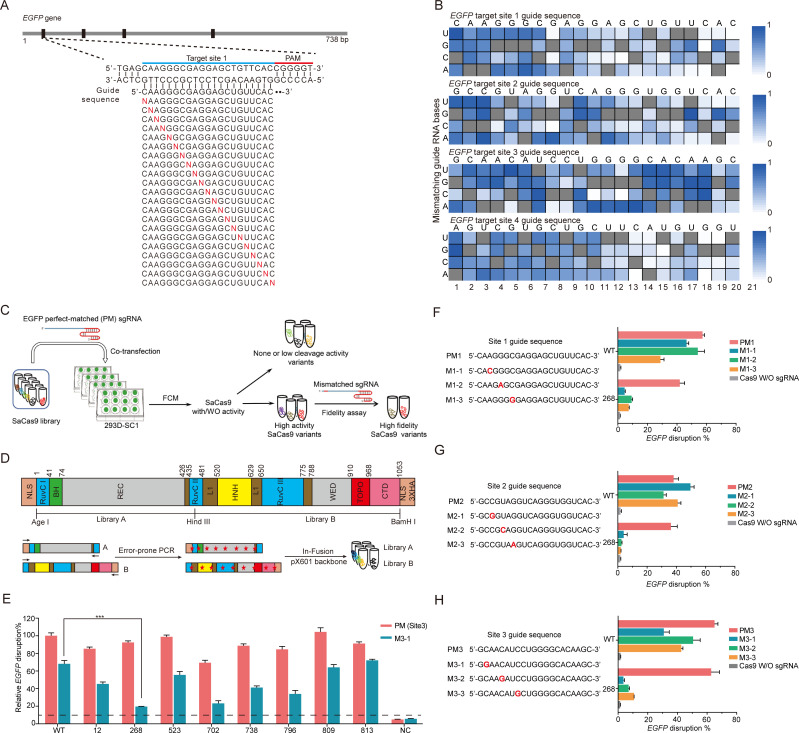
Library construction and screening of SaCas9-HF variants. (A) Schematic of the protocol for CRISPR-SaCas9-mediated gene editing of *EGFP*. (B) Heatmap represents the relative SaCas9 cleavage efficiency by 63 single-mutated and one perfect-matched sgRNA each for 4 *EGFP* target sites (*n* = 3). Nonmutated guide sequence is shown above and highlighted in gray squares in the heatmap. Relative *EGFP* disruption efficiencies (increasing from white to blue) are normalized to the nonmutated guide sequence. The data underlying this figure can be found in S1 Data. (C) The schematic of constructing two mutational SaCas9 libraries by error-prone PCR with in-fusion methods. “*” represents the mutation. (D) The schematic strategy of screening SaCas9-HF variants. (E) Eight variants were investigated with perfect-matched sgRNA site 3 sgRNA and single-nt mismatched sgRNAs site 3 sgRNA. Relative disruption efficiencies are normalized to the perfect-matched guide sequence with WT SaCas9; error bars, SEM; *n* = 3. (F–H) Validation of enhanced fidelity of Mut268 variant. For each site, WT SaCas9 and variants were assessed by *EGFP* disruption assay with a perfect-matched sgRNA or single-nt mismatched sgRNAs. The data underlying this figure can be found in S2 Data; error bars, SEM; *n* = 3. 268, Mut268; *EGFP*, enhanced green fluorescence protein; FCM, flow cytometry; M, mismatched; M3-1, single-nt mismatched sgRNA site 3; PM, perfect-matched; PM3, perfect-matched sgRNA site 3; SaCas9, *Staphylococcus aureus* Cas9; sgRNA, single-guide RNA; single-nt, single nucleotide; W/O, without; WT, wild-type.

For each of the 4 target sites, we generated a set of 63 different guide RNA sequences containing all possible single-nucleotide (single-nt) substitutions upstream of 5′-NNGRRT-3′ PAM (R:A/G, Fig 1A). The ability of these single-guide RNAs (sgRNAs) harboring single-nt mismatches to disrupt the target gene *EGFP* then served as a measure of the off-target effect. We found that single-nt mismatched sgRNAs, when used with WT SaCas9, achieved 7%–120% activity, compared with the perfect-matched sgRNA (set as 100%) with WT SaCas9 (Fig 1B). As with previous findings of SpCas9, SaCas9 tolerates single-nt mismatches in the PAM-distal region (5′ end) better than in the PAM-proximal region (3′ end) (Fig 1B) [10–12]. This demonstrated that our assay system can simultaneously measure on-target cleavage activity using the perfectly matched sgRNA as well as quantify off-target effects using the mismatched sgRNAs. Thus, this method allows for facile isolation of high-fidelity variants that also maintain robust on-target activities—a key challenge in previous efforts (Fig 1C).

### Library construction of engineered SaCas9 variants

To simplify library construction, we split the expression cassettes of SaCas9 and sgRNA from the all-in-one pX601 plasmid onto 2 separate plasmids that code for WT SaCas9 (empty pX601) and sgRNA targeting *EGFP* site 3 individually. We verified that co-transfection of the 2 vectors leads to efficient cleavage of *EGFP* (S1C Fig), while no activity has been found with other groups except the positive control (S1C Fig). Thus, SaCas9 and sgRNA could be expressed separately for efficient DNA cleavage. Therefore, we proceeded to introduce mutations into empty pX601 for the construction of the SaCas9 variant library.

We generated SaCas9 variants via random mutagenesis in SaCas9 coding sequence (Fig 1D and S2 Table). The coding sequence of SaCas9 was split into 2 segments demarcated by 3 unique restriction enzymes sites (AgeI, HindIII, and BamHI). Using error-prone PCR (Materials and Methods), we separately introduced mutations into the N- and C-terminal segments of Cas9 to form library A and library B, respectively. We obtained a total of 1,041 colonies and named them Mut1 through Mut1041, with 667 colonies in library A and 374 in library B. To estimate the rate of mutagenesis, we randomly picked 24 individual colonies from library A and found that 22 colonies contained 1 to 11 mutations and 2 colonies contained none (average mutation rate approximately 4.2 substitutions/kilobase, S3 Table). To estimate the library coverage, we randomly selected 18 additional colonies from library B and sequenced all 42 colonies from both libraries. Sequence analysis showed that two of them (2/42 = 4.8%) from library A and one of them (1/42 = 2.4%) from library B contained the WT coding sequence. The majority of the colonies (39/42 = 92.8%) harbored one or more mutations. Based on this sampling, the total library is estimated to contain over 900 individual variants.

### Screening of SaCas9-HF variants

To isolate high-fidelity, high-activity SaCas9 variants, we first eliminated low-activity variants one by one (Fig 1C). Using a perfect-matched sgRNA targeting site 3 (PM3), we found that 272 (28.1%) variants retained at least 70% of the WT activity (S2 Fig), which were subsequently subjected to fidelity measurements using a single-nt mismatched sgRNA (M3-1) (S3 Fig).

Twenty-two of the 272 variants exhibited improved fidelity against single-nt mismatches, most of which derived from library A. We then examined whether there are mutation hot spots in the SaCas9 coding sequence associated with improved fidelity. Interestingly, most variants were fairly evenly distributed across the entire open reading frame (S4A Fig), and the major mutation type is missense mutation (S4B Fig). Based on the fidelity (the ratio of cleavage efficiency of perfect-matched activity [PM3] to mismatched activity [M3-1]), we selected 8 variants from the pool of 22 for further testing and further narrowed down to 2 best behaved variants: Mut268 and Mut738 (Fig 1E).

### Validation of the high-fidelity phenotype

In comparing Mut268 and Mut738, Mut268 exhibited notably higher fidelity than Mut738 in the targeted site 3 with three different single-nt mismatched sgRNAs (S5 Fig). Hence, subsequent studies were focused on Mut268 using sites 1–3. Site 4 was excluded from further analysis as it is an inherently higher-fidelity site even by the WT SaCas9 (Fig 1B). Reassuringly, we found that Mut268 exhibited significantly enhanced fidelity in all 3 target sites (Fig 1F–1H and S6 Fig). These findings suggest that the mutation in Mut268 brings about across-the-board enhancements of cleavage fidelity in various sequence contexts, suggesting a fundamental change to the catalytic properties of the enzyme.

### Fidelity validation of Mut268 on endogenous sites with targeted deep sequencing

**Fig 2:**
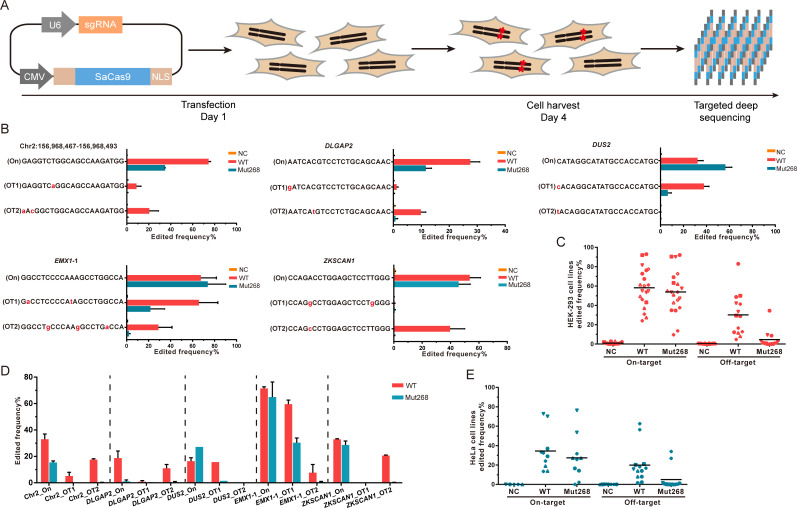
Fidelity validation of improved specificity for Mut268 at ten endogenous loci. (A) Illustration of CRISPR-SaCas9-mediated genome editing at endogenous genes. Ten endogenous-targeted sites were selected for fidelity test. (B) WT and Mut268 mediated cleavage at on-target and predicted off-target sites. Cleavage efficiency was examined by targeted deep sequencing. Edited products containing nucleotides substitution, in/del were analyzed. Mismatched nucleotides in predicted off-target sites are highlighted in red. The data underlying this figure can be found in S3 Data. Error bars, SEM; *n* = 2. (C) Comparison of WT and Mut268 for the enhanced specificity in HEK-293 cell lines. All 10 on-target sites were plotted to compare the on-target activity between WT and Mut268. Off-target sites edited by WT SaCas9 with obvious off-target effects and Mut268 were plotted. Sample size of 10 in on-target sites and 7 in off-target sites. (D) WT and Mut268 mediated cleavage at on-target and predicted off-target sites in HeLa cells; error bars, SEM; *n* = 2. (E) Comparison of WT and Mut268 for the enhanced specificity in HeLa cells. in/del, insertion/deletion; NC, negative control (without transfection of Cas9-expression plasmid); “On,” on-target; OT, predicted off-target; SaCas9, *Staphylococcus aureus* Cas9; WT, wild-type.

To systematically evaluate the activity and fidelity of Mut268 SaCas9 at endogenous chromosomal sites in addition to *EGFP*, we compared the specificities of WT and Mut268 SaCas9 at 10 endogenous sites using targeted deep sequencing and T7EI (T7 Endonuclease I) assay (Fig 2A and 2B, S7 Fig and S8A–S8C Fig). We observed a dramatic different cleavage behavior between the Mut268 and WT SaCas9 at the targets of chr2:156,968,467–156,968,493, *DLGAP2*, *DUS2*, *EMX1-*1, and *ZKSCAN1* (Fig 2B). In HEK-293 cells, Mut268 generated low levels of nonspecific editing at predicted off-target sites, and an improvement of approximately 2- to 93-fold compared to WT SaCas9 (S8B Fig). Strikingly, Mut268 achieved 15.8- and 93-fold improvement in fidelity at *EMX1-1*_OT2 and *ZKSCAN1_*OT2 sites, respectively. Of note, reduction of on-target activity was observed at certain sites (Chromosome 2: 156,968,467–156,968,493 and *DLGAP2* site), whereas on-target activity at *DUS2* was enhanced (Fig 2B). The average on-target activity of Mut268 at these 10 sites in HEK-293 cells was about 92.6% of that of WT (Fig 2C). We also performed fidelity tests in 2 additional cell lines, namely HeLa and HT-1080 cells, and also observed fidelity improvement of Mut268 compared with WT SaCas9 (Fig 2D and 2E and S8D Fig). Taken together, Mut268 SaCas9 possesses significantly reduced off-target effects across a number of human cell lines and in diverse genomic contexts—suggesting its general utility.

### Unbiased off-target screening for Mut268 fidelity

**Fig 3:**
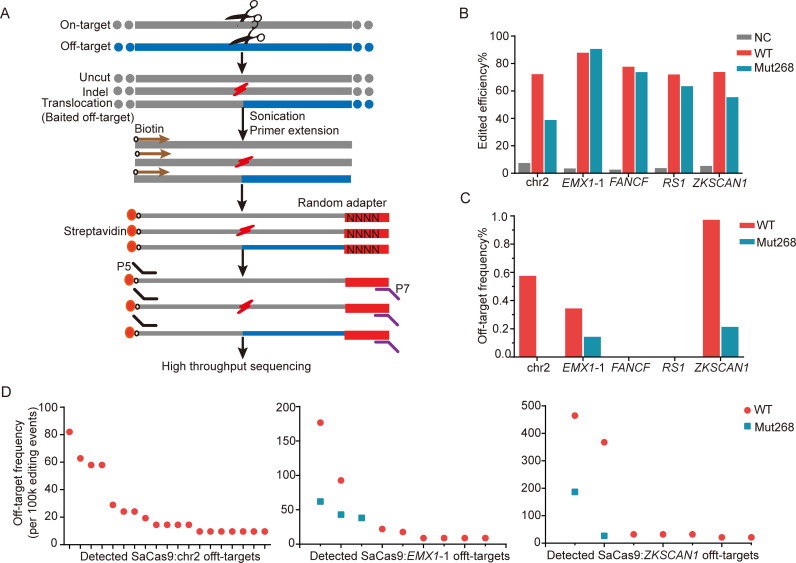
Mut268 possesses lower off-target effects at genome-wide levels. (A) Schematic of PEM-seq. (B) Edited efficiency for WT SaCas9 and Mut268 detected by PEM-seq. chr2 represents chr2:156,968,467–156,968,493 locus. (C) Off-target frequency for WT SaCas9 and Mut268 detected with PEM-seq. (D) Scatter plot of indicated off-target hotspots for WT and Mut268. y-Axis showed frequency of each hotspot per 100,000 editing events (in/dels plus translocation). in/del, insertion or deletion; PEM-seq, primer-extension-mediated sequencing; SaCas9, *Staphylococcus aureus* Cas9; WT, wild-type.

Several methods have been developed for detection of Cas9-triggered off-target effects [8,9,33,34]. To evaluate fidelity of Mut268 at the genome-wide level, we performed primer-extension-mediated sequencing (PEM-seq) (Fig 3A) [34]. On-target activity and fidelity of Mut268 at 5 endogenous sites in HEK-293 cells were evaluated. We observed that the in/dels caused by Mut268 were 38%–90%, compared to 65%–87% by WT SaCas9 (Fig 3B). This finding is largely consistent with the results of targeted deep sequencing analysis (Fig 2B). Regarding fidelity, Mut268 exhibited significantly lower levels of nonspecific editing genome wide (0.0%–0.21%) compared with the WT SaCas9 (0.0%–0.97%) (Fig 3C). In addition, many off-target sites detected in the WT SaCas9 are not detected by Mut268 (Fig 3D). Therefore, all these results further confirmed that Mut268 possesses higher fidelity than WT SaCas9 in broad contexts while maintaining WT-like on-target activity.

### Key residue for Mut268 fidelity

**Fig 4:**
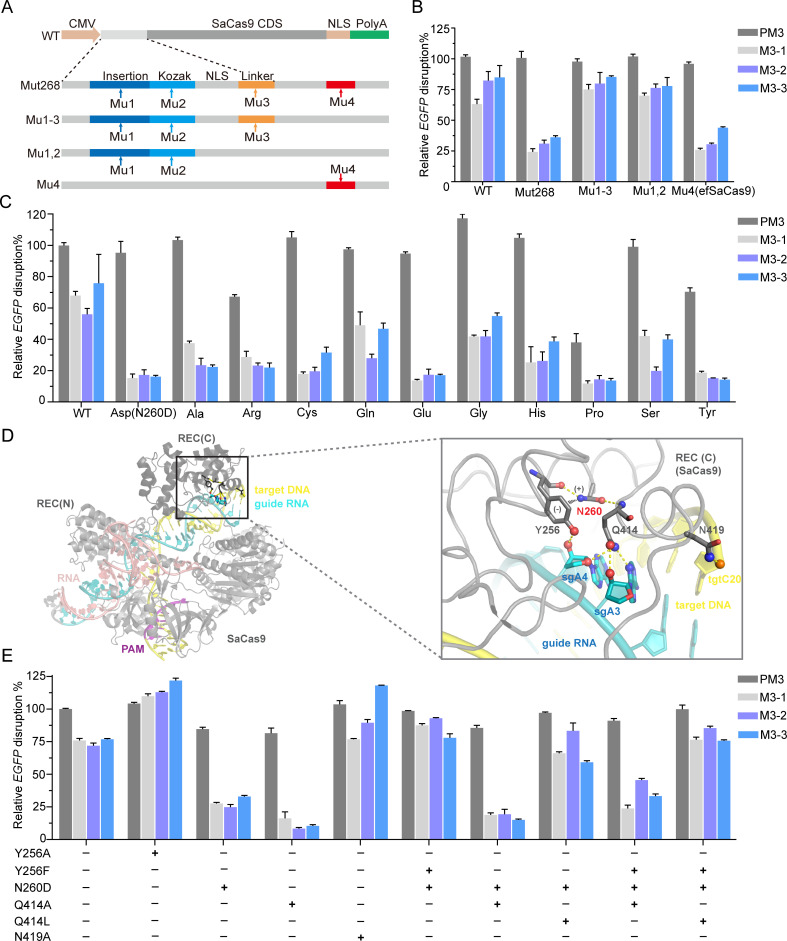
Structural insights of enhanced fidelity. (A) Strategy for mapping the responsible mutation(s). (B) Relative *EGFP* disruption of site 3 with variants harboring different mutation(s). Relative disruption efficiencies are normalized to the perfect-matched sgRNA target site 3 recognized by WT SaCas9; error bars, SEM; *n* = 3. (C) Effects of single amino-acid substitutions of N260 on SaCas9 editing activity and fidelity. (D) Structural context of efSaCas9. Left panel: Overall structure of a ternary complex formed by SaCas9 (grey), sgRNA (cyan), and target DNA (yellow). The REC1 and REC3 domains of the REC lobe are indicated. The PAM sequence is in magenta. Right panel: Zoomed-in view of the boxed region in (A), showing the interaction between the REC3 domain with the heteroduplex. Hydrogen bonds are indicated by yellow dotted lines. The cation interaction between N260 and Y256 are indicated by the partial charges and black dotted lines (PDB:5CZZ). (E) Fidelity comparisons of structure-guided additional SaCas9 variants with perfect-matched sgRNA target site 3 and single-nt mismatched sgRNAs; error bars, SEM; *n* = 3. efSaCas9, enhanced-fidelity SaCas9; M, single-nt mismatched sgRNA; PAM, protospacer adjacent motif; PM3, perfect-matched sgRNA target site 3; REC,; SaCas9, *Staphylococcus aureus* Cas9; sgRNA, single-guide RNA; single-nt, single nucleotide; WT, wild type.

To map the exact sequence changes in Mut268, we sequenced its entire SaCas9 expression cassette (promoter, coding sequence and Poly (A)) (Fig 4A). We found 4 mutation sites in the sequence, namely Mu1, Mu2, Mu3, and Mu4 (Fig 4A). Specifically, Mu1 and Mu2 were in/del mutations at the Kozak sequence, which resulted from mutagenesis in the region between the cytomegalovirus (CMV) promoter and Kozak sequence. Mu3 is a linker G>C substitution (corresponding residue change from A [alanine] to P [proline]), located between the SV40 nuclear localization signal (NLS) and SaCas9 coding sequence. Finally, Mu4 is a c.778A>G (N260D) substitution, located at the REC3 domain of SaCas9. When we reverted the Mu4 site back to the WT, the enhanced fidelity was abolished (Fig 4B, Mu1-3 and Mu1,2), which suggested that the Mu4 mutation is the driver of the enhanced fidelity of Mut268. We also found that mutant harboring only Mu4 (N260D) but not Mu1-3 had almost the same enhanced fidelity as Mut268 (has Mu1-4). We also tested the expression levels of WT SaCas9 and the high-fidelity variants, which revealed that there was no appreciable change in the level of protein expression with the mutation (S10 Fig). To better understand the role of N260, we generated a panel of point mutants whereby N260 was changed to a number of other side chains (Fig 4C, S11 Fig). Notably, we found that all of the variants except for the N260P mutant retained the majority of the cleavage activity and exhibited enhanced fidelity compared with that of the WT. Interestingly, when N260 was changed to Q, which has a similar side-chain as N (WT) but with one additional carbon in the side chain, it had similar cleavage activity but increased fidelity. As for N260E, we expected it to be similar to the Mu4 mutation (N260D), because both N260E and N260D installed a negatively charged amino acid at physiological pH (Fig 4C). The results showed that this was indeed the case. Collectively, the data suggested that the loss of the asparagine side chain of N260 was responsible for the increased fidelity of Mut268. Hence, we named the N260D variant enhanced-fidelity SaCas9 (efSaCas9).

### Structural insights into the enhanced fidelity of efSaCas9

To clarify the mechanism whereby the N260D substitution endowed efSaCas9 with enhanced fidelity, we analyzed the protein–protein and protein–nucleic-acid interactions identified in co-crystal structures [28]. SpCas9 and SaCas9 share a conserved bilobed architecture consisting of a recognition (REC) lobe and a nuclease (NUC) lobe, which together snugly envelope the target DNA-guide RNA heteroduplex (Fig 4D). The N260 substitution maps to the REC3 domain of the REC lobe. Remarkably, we found that N260 resides at the nexus of a network of stabilizing interactions (Fig 4D). The δ amine of N260 positions the aromatic side chain of Y256 through a cation (amine)–π interaction, to enable a single Y256 contact to nucleotide 4 of sgRNA. Importantly, the carbonyl group of N260 caps a short α-helix (Q414-L421) that resides over the major groove of the heteroduplex. N260 appears to position this α-helix to enable extensive contacts between SaCas9 and the heteroduplex.

To test the hypothesis that N260 bridges a network of interactions to affect the enzyme’s grip on the DNA-RNA heteroduplex, we introduced additional substitutions at Y256, Q414, and N419 positions (S2 Table). Neither Y256F nor Y256A substitution substantially altered the cleavage activity or fidelity of SaCas9 (Fig 4E). Remarkably, a Q414A variant exhibited even higher fidelity than N260D while retaining most on-target activity. Specifically, Q414A had activities of 16.3%, 8.3%, and 10.4% on M3-1 through M3-3 sgRNA, appreciably lower than the 27.7%, 24.8%, and 32.9% of N260D (Fig 4E). Interestingly, a variant with Q414L and N260D double substitutions (Q414L + N260D) exhibited an intermediate phenotype between the WT and Q414A variant. A possible explanation is that an isobutyl side chain of leucine at the 414 position can stack with the nucleobase of SgA4 (A4 of sgRNA) to stabilize it, and thus partially compensate for the removal of the 3 hydrogen bonds normally engaged by Q414 (Fig 4D).

Then we sought to combine these substitutions to ask whether these residues act collaboratively or independently. When Q414A was introduced into the N260D variant (N260D + Q414A), the double-mutation variant exhibited a similar phenotype (activity of 18.9% at M3-1, 19.4% at M3-2, 15.1% at M3-3) as N260D or Q414A alone. This nonadditive effect suggests that Q414 and N260 act through a common mechanism. Moreover, we found that the effects of Q414 substitutions overrode those of N260. In the context of the Q414A or Q414L variant, the introduction of N260D no longer had any phenotype (Fig 4E). These findings demonstrate that N260 likely functions through Q414, which makes direct, functionally important contacts to the 5′ region of the guide RNA.

Taken together, our data suggest that N260 substitutions in REC3 domain likely weaken the clamp–duplex interaction of Cas9 with the heteroduplex, thus amplifying the selectivity for heteroduplexes with perfect-matched 5' region and leading to enhanced fidelity.

### Fidelity estimates of catalytically inactive efSaCas9 by transcriptional activation and chromatin immunoprecipitation sequencing analyses

Finally, we sought to explore whether efSaCas9 still possesses higher fidelity in a catalytically inactive form (S12 Fig) [35]. With the 5′ mismatches or 3′ mismatches in the sgRNA, we did not observe significant differences in the expression of mCherry between dSaCas9-VPR and defSaCas9-VPR. The results of chromatin immunoprecipitation sequencing (ChIP-seq) also showed that efSaCas9 and WT SaCas9 have similar DNA binding activity with the mismatched DNA/RNA substrates (S13 Fig). The observations that a catalytically inactive form of efSaCas9 does not decrease off-target effect indicate that efSaCas9’s enhanced fidelity is associated with its catalytic cycle, in which kinetic effects (e.g., faster dissociation or reduced dwell time on mismatched heteroduplexes) may be responsible for the observed fidelity enhancement.

## Discussion

Off-target effects of CRISPR-Cas9 mediated genome editing is a major concern for therapeutic applications. Using an in vivo *EGFP* reporter system, we first delineated off-target effect of WT SaCas9 at the single-nt level (Fig 1B). Indeed, we observed significant off-target cleavage activities of WT SaCas9 using certain mismatched sgRNA (Fig 1G). To mitigate these issues with WT SaCas9, we described a directional screening system that can efficiently select SaCas9 variants with desired properties and traits. As for the screening strategy, our human cell-based *EGFP* reporter system is efficient in rapidly assessing the activity and fidelity of variants quantitatively and reproducibly. We previously took advantage of this system to compare the DNA cleavage activity of CRISPR-SpCas9 with noncanonical PAM sequences [29]. Also, we found that FnCpf1 (FnCas12a) possesses genome editing activity in human cells with this system [30–32]. In the present study, we identified efSaCas9 and also obtained additional variants—i.e., Mut 738—which may be further engineered to increase its fidelity.

While our study was being prepared for publication, Tan and colleagues reported the rational design of an SaCas9-HF based on the structure of SaCas9 [36], which is a different strategy from our direct screening in human cells. Genome-wide, unbiased identification of dsbs enabled by sequencing (GUIDE-seq) and targeted deep sequencing results showed that SaCas9-HF has 79% on-target activity of WT. With Mut268, we also observed partial loss of on-target efficiency at certain sites (Fig 2C–2E). It is reported that high-fidelity Cas9s (i.e., SpCas9-HF1, eSpCas9, and xCas9) generally exhibit reduced cleavage activity at certain sites [36,37]. Further study will be performed to understand and boost on-target activity at low-efficiency target sites and to compare the fidelity of efSaCas9 with SaCas9-HF.

To broadly evaluate genome-wide off-target effect of Cas9, several methods (GUIDE-seq, in vitro Cas9-digested whole-genome sequencing [Digenome-seq], and circularization for in vitro reporting of cleavage effects by sequencing [CIRCLE-seq]) have been developed [8,9,33]. However, these methods are unable to directly determine the on-target editing efficiency of CRISPR/Cas in vivo. PEM-seq is a modified method of linear amplification–mediated high-throughput genome-wide sequencing (LAM-HTGTS) [34,38], which provides comprehensive information of CRISPR/Cas9 editing events, especially chromosome translocation. Using PEM-seq, our results clearly showed that Mut268-triggered off-target frequency was reduced compared with the WT SaCas9 (Fig 3B–3D).

Recent studies revealed allosteric linkages between REC3 and HNH domains in SpCas9, and mutagenesis in the REC lobe could confer higher fidelity to SpCas9 [22,25,39]. To further understand the enhanced fidelity of efSaCas9 (N260D), we performed mutational analyses and confirmed the importance of the REC3 domain of SaCas9 in providing accurate targeting (Fig 4C–4E). However, it remains unclear whether the stability of the REC3 clamp-duplex, the dwell time of SaCas9 on mismatched heteroduplex, or the rates of conformational changes associated with the catalytic cycle primarily contribute to fidelity [22]. Interestingly, we found that efSaCas9 does not confer increased fidelity against mismatches near the 3' end of the sgRNA (S15 Fig). This observation can be explained by the fact that N260 is located more than 55 Å away from this region and is unable to exert long-range effects. Instead, the duplex near the 3' end of sgRNA is recognized by the REC1 domain, the bridge helix, and the phosphate lock loop (Fig 4D). Further mutations on the coding sequence of REC1 may lead to the enhancement of its specificity in the PAM-proximal region. Interestingly, comparative sequence analysis of several Cas9 enzymes showed that the N260 and Q414 residues of SaCas9 are not conserved in *Campylobacter jejuni* Cas9 (CjCas9), *Neisseria meningitidis* Cas9 (NmCas9), and SpCas9 but are identical with those in *Streptococcus thermophilus* Cas9 (St1Cas9) (S14 Fig). Based on this, equivalent substitutions to N260D and Q414A in St1Cas9 may also result in the desirable enhanced fidelity trait.

Based on the results of DNA cleavage, transcriptional activation, and ChIP-Seq (Fig 1F–1H, Fig 2B–2E, S12 Fig and S13 Fig), we favor the following model for the enhanced fidelity of efSaCas9. It could be due to an altered threshold of conformational change, rather than reduced binding efficiency (S12B and S12C Fig). Specifically, we propose that N260 substitution in REC3 domain delays the activation of the HNH domain when SaCas9 binds to off-target DNA substrates (S12C Fig), similar to the reported SaCas9-HF-like evoSpCas9 and HypaCas9 [22,25]. Further studies will be required to ascertain the precise mechanisms by which efSaCas9 achieves its high specificity.

Taken together, in the present study, we delineated off-target effect of WT SaCas9 and, using a directional screening system in human cells, we identified efSaCas9, a high-fidelity, high-activity variant that could be harnessed for safe and reliable genome editing. The rapid screening and evaluation system is further broadly applicable for the isolation of new variants with other desirable traits in other Cas9 systems.

## Materials and methods

### Plasmids and DNA analysis

The lentiviral vector plasmid pSIN-EGFP containing an *EGFP* gene, *IRES* and *Puromycin* gene was generated from pSIN-EF2-Lin28-Puro (Addgene plasmid #16580) using EcoRI and BamHI restriction enzyme sites. SaCas9 plasmid was a gift from Feng Zhang (Addgene plasmid #61591); VPR expression plasmid (GP230) and mCherry reporter plasmid (ZP30) were gifts from Dr. Yang, Hui (Shanghai Institutes for Biological Sciences, CAS). CRISPR-Cas9 plasmids were constructed as described online (http://www.genome-engineering.org/crispr/). The oligonucleotide sequences for sgRNA construction are summarized in S2 Table. Plasmid DNA and genomic DNA were isolated by standard techniques. The DNA sequencing confirmed the desired specific sequence in the constructs.

### Cells and cell culture

HEK-293 cells were obtained from ATCC (CAT#CRL-1573) and grown at 37°C in 5% CO_2_ in Dulbecco’s Modified Eagle Medium (Life Technologies, Carlsbad, CA), 10% heat-inactivated fetal bovine serum, penicillin/streptomycin. HEK-293 cells expressing *EGFP* were described previously [29]. Drug-resistant single colonies of transduced HEK-293 cells were isolated and named 293-SC1. To maintain EGFP expression, the medium for 293-SC1 culture includes puromycin.

### Construction of SaCas9 library

The library was generated by error-prone PCR (primers sequence in S1 Table). Specifically, Plasmid (pX601) harboring SaCas9 coding sequence was digested with AgeI/HindIII or HindIII/BamHI, respectively. The AgeI/HindIII or HindIII/BamHI fragments were mutated by random mutagenesis kit (CAT#101005, TIANDZ) and then purified, in-fusion with linearized pX601 backbone without the corresponding fragment. The individual colonies from LB plate were then manually picked. The plasmids from individual colonies were isolated. The concentration of each plasmid was adjusted as 100 ng/μL.

### Targeted deep sequencing

Off-target sites (S1 Table) were predicted by Cas-OFFinder software [40], and off-target sites with fewer than 5 mismatched nucleotides were screened. In addition, the off-target sites of OT1 and OT2 for *EMX1*_1 were reported [5]. Off-target sites for chr2:156,968,467–156,968,493, *DLGAP2*, and *DUS2* were screened by algorithm for fewer than 2 mismatched nucleotides in whole genome. Targeted deep sequencing experiments were performed with WT SaCas9 and Mut268 for different loci at human genome. Briefly, 1.8 × 10^5^ HEK-293 cells were transfected with 750 ng of all-in-one expression plasmids. Seventy-two hours after transfection, genomic DNA was extracted using standard phenol/chloroform extraction protocols. For the construction of the NGS library, the primary PCR was performed to amplify 100 to 230 bp on/off-target sites from approximately 60 ng of genomic DNA using Phanta Super-Fidelity DNA Polymerase (Vazyme Biotech Co., Ltd). The secondary amplification was to fix barcodes, index, and adaptor sequences into the primary PCR products (S2 Table). Amplification products were purified (Thermo Fisher Scientific) and pooled into one tube. After the removal of adaptors and low-quality reads, paired-end reads were merged and then mapped to the template. Base substitution and in/del were analyzed using open-sourced “CRISPResso” software (version 1.0.10) with read quality above Q30 [41].

### PEM-seq for SaCas9

The protocol has been previously described [42]. Specifically, 3.2 μg pX601 plasmids were transfected into HEK-293 cells in 6-cm dishes. Cell were harvested 48 h post transfection followed by standard PEM-seq procedure. Hiseq reads were processed by “SuperQ” pipeline, and off-target hotspots were identified by “MACS2” *callpeaks* mode. MACS2 results were further filtered to remove sites with fewer than 2 junctions and no target site-similar sequence by “Bedtools” and “Needle.”

### T7EI assay for gene editing

Briefly, 293-SC1 were plated at a density of 1.8 × 10^5^ cells per well in a 12-well plate on day 0 and transfected with 750 ng CRISPR-SaCas9-sgRNA plasmids with Turbofect on day 1. Fresh medium was added to the transfected 293-SC1 cells on day 2. Cells were harvested on day 3. For T7EI assay, 150 ng purified PCR products were mixed with 1.5 μL 10× NEB#2 buffer and ultrapure water to a final volume of 14.5 μL and were subjected to re-annealing process to enable heteroduplex formation. After re-annealing, products were treated with 0.5 μL T7 Endonuclease I for 45 min.

### Transcriptional activation assay

The ChIP-seq assay was performed as described previously [7]. For the fluorescence reporter assay, 1.0 × 10^5^ HEK-293 cells of each well (24-well plates) were seeded on day 0. On day 1, each well of was transfected with 375 ng of dCas9-VPR plasmid, 150 ng of plasmid containing sgRNA, and 250 ng of miniCMV-mCherry plasmid. Fresh medium was added to the transfected cells on day 2. Cells were harvested for FCM on day 3.

### Western blotting

HEK-293 cells were plated at a density of 5.0 × 10^5^ cells per well in a 6-well plate on day 0 and transfected with 1.5 μg plasmids (pX601, Mut268, and efSaCas9) via TurboFect transfection reagent on day 1. Fresh medium was added after 12 h. Cells were harvested on day 3. Proteins were analyzed on SDS-PAGE after quantification. Membranes were blotted with antibodies directed at the following proteins: HA (Mouse-1F5C6, Proteintech Cat#66006-2-Ig, 1:2,500 dilution) and β-Actin (Mouse-8H10D10, Cell Signaling Technology Cat#3700, 1:1,000). An HRP-conjugated secondary antibody (Goat, Abcam Cat#ab97023, 1:5,000) was used for chemiluminescent detection.

## Supporting information

S1 Fig CRISPR/SaCas9-mediated *EGFP* disruption. (A) The effect of plasmid amount for CRISPR/SaCas9-mediated *EGFP* disruption targeting site 3. (B) *EGFP* disruption efficiency at 5 different sites with CRISPR/SaCas9. (C) pX601 (CRISPR/SaCas9, WT SaCas9) with perfect-matched sgRNA3 (PM3, site 3) induced *EGFP* inactivation. pX601 empty vector plus sgRNA could induce *EGFP* inactivation (the first line) or plasmid with pX601-sgRNA (the third line, the vector has 2 expression cassettes, one for SaCas9 and the other for sgRNA). pX601ΔCas9 represents the pX601 plasmid without SaCas9 coding sequence.
(TIF)

S2 Fig On-target activities of SaCas9 mutants. Cleavage with SaCas9 mutants plus perfect-matched sgRNA targeting *EGFP* site 3 (PM3), we found 272 mutants have activity above the cut-off value (≥70% activity of WT SaCas9). Here we only showed these mutants.
(TIF)

S3 Fig Fidelity of SaCas9 mutants. SaCas9 mutants’ activity and fidelity were investigated with perfect-matched sgRNA (PM3) and single-nt mismatched sgRNA (M3-1), respectively. The x-axis represents the ratio of *EGFP* disruption percentage by M3-1 over PM3 sgRNA. Lower bars indicate higher fidelity.
(TIF)

S4 Fig Mutation information for SaCas9 variants. (A) Distribution and frequency of amino acid substitutions of 22 SaCas9-HF variants. Each mutation from the SaCas9 variants was mapped to the coding sequence of SaCas9. (B) Pie chart for the mutation type of 22 SaCas9-HF variants.
(TIF)

S5 Fig Fidelity investigation of SaCas9 mutants. Two SaCas9 mutants’ activity and fidelity were investigated.
(TIF)

S6 Fig Fidelity test of WT SaCas9 and Mut268 via FCM. sgRNA was designed to target site 1, and mismatched sgRNA (M1-2) was used to test the fidelity of SaCas9. FCM results for Fig 1F.
(TIF)

S7 Fig T7EI assay for WT and Mut268 mediated cleavage at predicted off-target loci in HEK-293 cell lines. Red arrows represent cleaved bands. The percentage represents the cut efficiency. NC represents negative control.
(TIF)

S8 Fig Enhanced fidelity of Mut268. (A) WT and Mut268 mediated cleavage at on target (On) and predicted off target (OT) sites measured by targeted deep sequencing. (B) On-/off-target ratios were calculated from the data in Fig 2B. (C) T7EI assay for the specificity of Mut268 at *EMX1*-1 site. (D) WT and Mut268 mediated cleavage in HT-1080 cell lines.
(TIF)

S9 Fig Genetic information of Mut268. DNA sequencing analysis of Mut268 revealed 4 mutations (Mu1–Mu4). WT and 268 represent the WT SaCas9 and Mut268, respectively.
(TIF)

S10 Fig Expression level for WT SaCas9 and high-fidelity variants. HA-tagged SaCas9 and its variants were analyzed by western blotting using the indicated antibodies. CTRL, HEK-293 cells without transfection; WT, wild-type SaCas9 (pX601); Mut268, Mut268 variant; efSaCas9, N260D variant.
(TIF)

S11 Fig Predicted structures of mutant SaCas9 harboring single substitutions at the 260 position. (A) Hydrogen bonding and cation-π interactions mediated by N260 in WT ScCas9. PDB ID:5CZZ. (B–K) Ten lowest-energy structures simulated of SaCas9 mutants by Rosetta Backrub: N260D (B), N260A (C), N260R (D), N260Q (E), N260E (F), N260G (G), N260H (H), N260S (J), and N260Y (K). Only the positions of the side chains of Y256, 260, and Q414 (green sticks) are shown for clarity. N260C simulation was not performed, as cysteine simulations are presently not available through Rosetta Backrub.
(TIF)

S12 Fig Transcriptional activation assay of efSaCas9. (A) Schematic representation of miniCMV-mCherry based transcriptional activator reporter. Upon binding of dSaCas9-VPR to miniCMV promoter, mCherry expression is activated. (B) mCherry activation was measured in HEK-293 cells transfected with dSaCas9-WT or defSaCas9 based transcriptional activators plus perfectly matched sgRNA or mismatched sgRNAs, as indicated; error bars, SEM; *n* = 3. (C) Model for enhanced fidelity of efSaCas9. Mutation in REC lobe may increases the threshold for HNH activation and cleavage when SaCas9 targets the mismatched RNA–DNA heteroduplex.
(TIF)

S13 Fig Fidelity investigation of efSaCas9 with ChIP-seq. Primers for qPCR were in supplementary S2 Table. Off target (OT) sites were predicted and measured by qPCR.
(TIF)

S14 Fig Analysis of Cas proteins. (A) Phylogenetic tree of Cas proteins. Phylogenetic tree was generated with COBALT software. (B) Alignment results of 5 Cas9 proteins. (C) Part of alignment results. Highly conserved sequences are shown in red. The N260 and Q414 residues of SaCas9 are marked by arrows.
(TIF)

S15 Fig Fidelity of SaCas9 mutants against mismatches near the 3' end of sgRNA. Fidelity comparisons of structure-guided additional SaCas9 mutants with perfect-matched sgRNA 3 (PM3) and corresponding single-nt mismatched sgRNAs (M); error bars, SEM; *n* = 3. Relative disruption efficiencies are normalized to perfect-matched sgRNA of WT SaCas9.
(TIF)

S1 Table Target sites used in this study. (XLSX)

S2 Table Primers used in this study. (XLSX)

S3 Table Mutagenesis rate of library A using Sanger sequencing. (XLSX)

S1 Data Relative SaCas9 cleavage efficiency by 63 single-mutated and one perfect-matched sgRNA each for four EGFP target sites. (XLSX)

S2 Data FCM results for enhanced fidelity of Mut268 variant and WT SaCas9. (XLSX)

S3 Data Detected reads for targeted deep sequencing. (XLSX)

## Abbreviations

AAV: adeno-associated virus
ChIP-seq: chromatin immunoprecipitation sequencing
CIRCLE-seq: circularization for in vitro reporting of cleavage effects by sequencing
CjCas9: *Campylobacter jejuni* Cas9
CMV: cytomegalovirus
Digenome-seq: in vitro Cas9-digested whole-genome sequencing
efSaCas9: enhanced-fidelity SaCas9
EGFP: enhanced green fluorescence protein
FCM: flow cytometry
GUIDE-seq: genome-wide, unbiased identification of dsbs enabled by sequencing
in/del: insertion or deletion
LAM-HTGTS: linear amplification–mediated high-throughput genome-wide sequencing
M3-1: single-nt mismatched sgRNA site 3
NmCas9: *Neisseria meningitidis* Cas9
NLS: nuclear localization signal
PAM: protospacer adjacent motif
PEM-seq: primer-extension-mediated sequencing
PM3: perfect-matched sgRNA site 3
RNP: ribonucleoprotein
SaCas9: Staphylococcus aureus Cas9
SaCas9-HF: high-fidelity SaCas9
sgRNA: single-guide RNA
single-nt: single nucleotide
SpCas9: Streptococcus pyogenes Cas9
St1Cas9: *Streptococcus thermophilus* Cas9
WT: wild type
