# Supplementary figures and images for: High-fidelity SaCas9 identified by directional screening in human cells
Source: PLoS Biol. 2020 Jul 9;18(7):e3000747. doi: 10.1371/journal.pbio.3000747 (PMC7347106; doi:10.1371/journal.pbio.3000747)

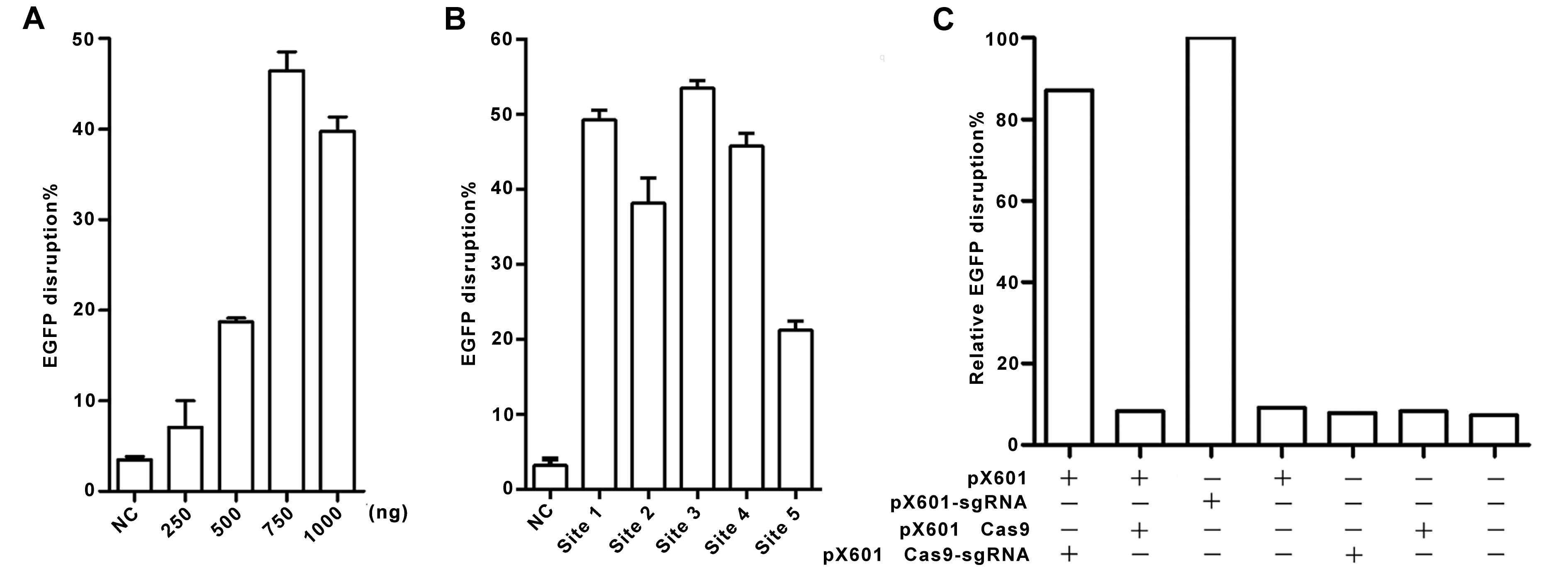

Supplement: S1 Fig — (A) The effect of plasmid amount for CRISPR/SaCas9-mediated EGFP disruption targeting site 3. (B) EGFP disruption efficiency at 5 different sites with CRISPR/SaCas9. (C) pX601 (CRISPR/SaCas9, WT SaCas9) with perfect-matched sgRNA3 (PM3, site 3) induced EGFP inactivation. pX601 empty vector plus sgRNA could induce EGFP inactivation (the first line) or plasmid with pX601-sgRNA (the third line, the vector has 2 expression cassettes, one for SaCas9 and the other for sgRNA). pX601ΔCas9 represents the pX601 plasmid without SaCas9 coding sequence. (TIF) [file pbio.3000747.s001.tif]

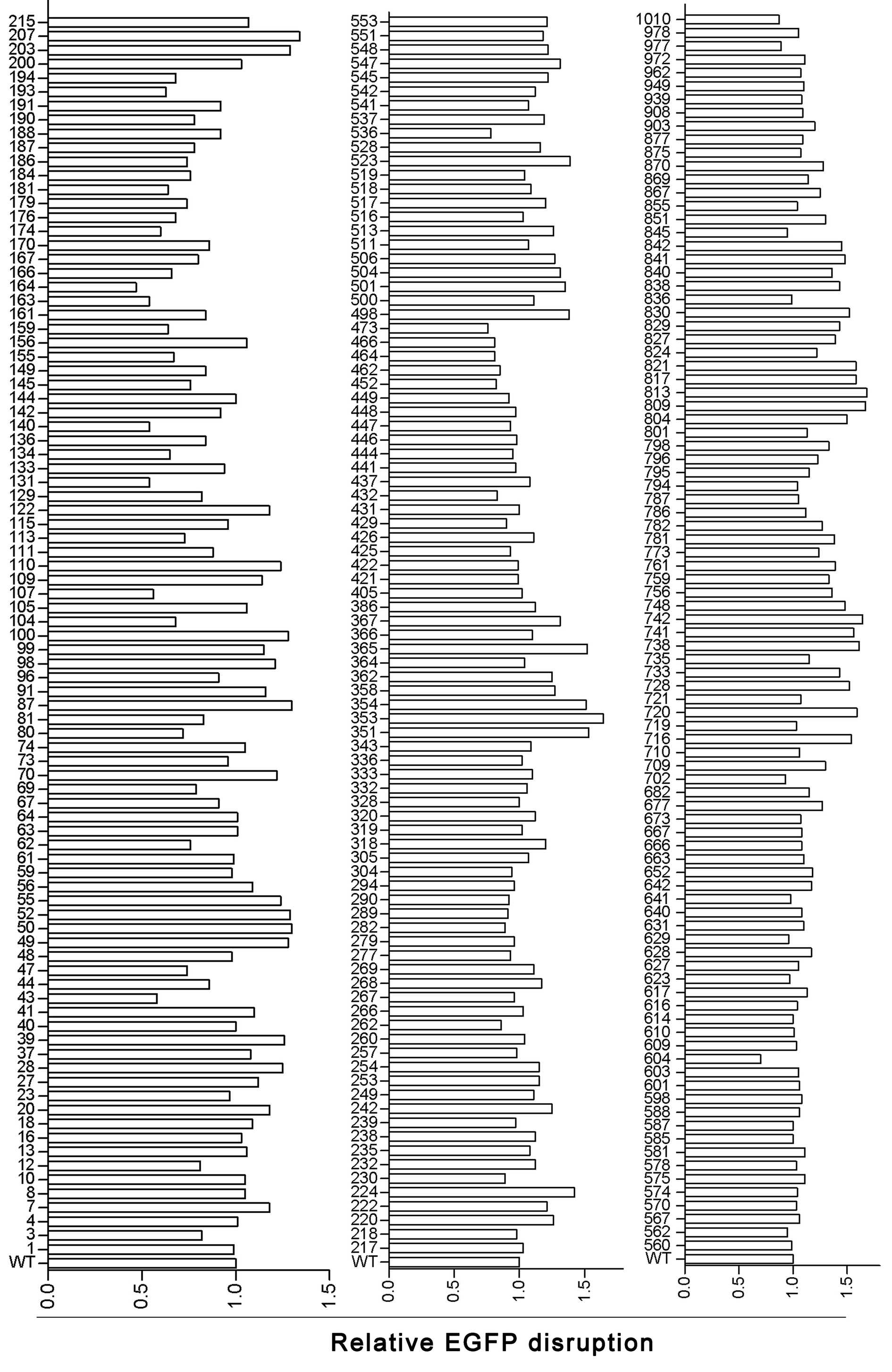

Supplement: S2 Fig — Cleavage with SaCas9 mutants plus perfect-matched sgRNA targeting EGFP site 3 (PM3), we found 272 mutants have activity above the cut-off value (≥70% activity of WT SaCas9). Here we only showed these mutants. (TIF) [file pbio.3000747.s002.tif]

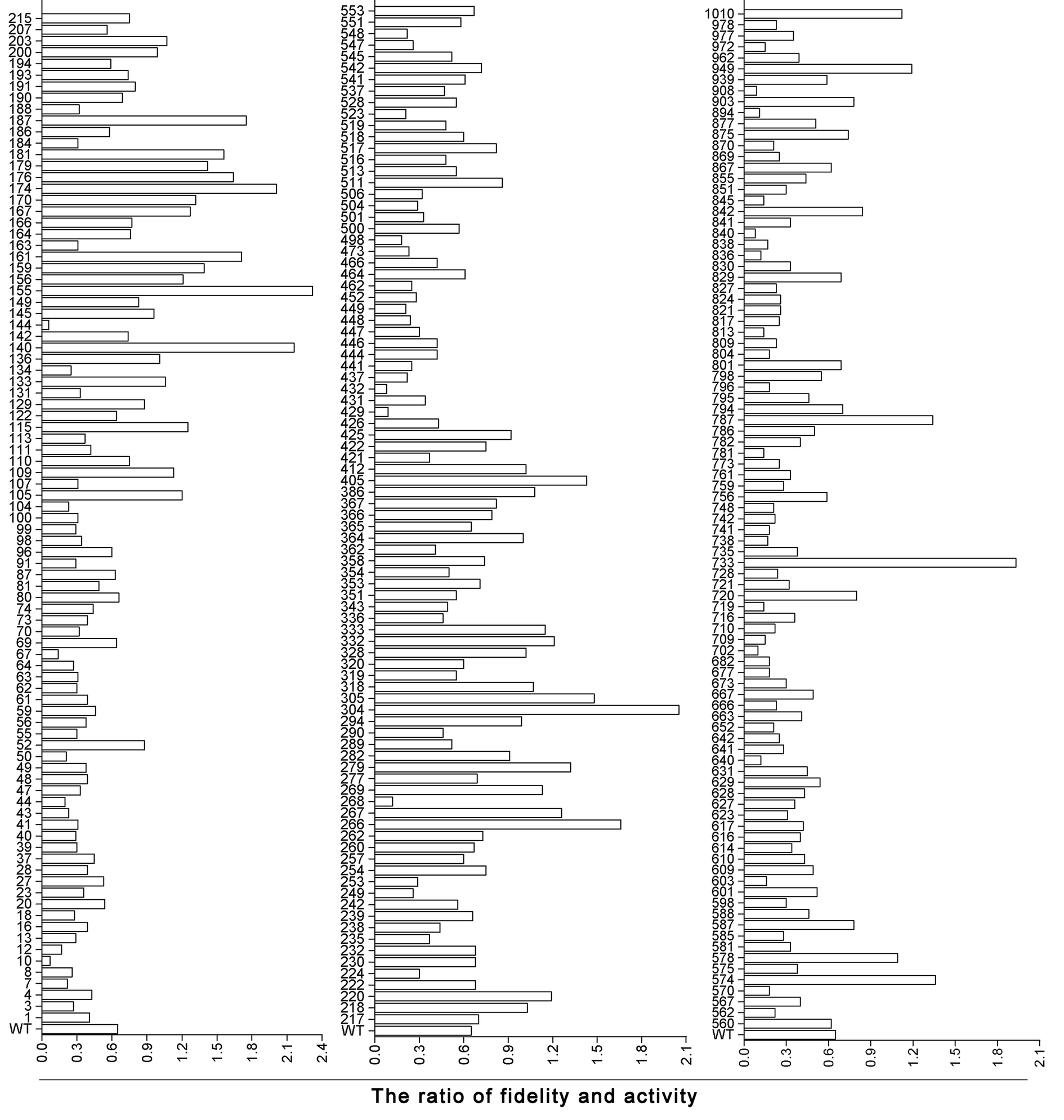

Supplement: S3 Fig — SaCas9 mutants’ activity and fidelity were investigated with perfect-matched sgRNA (PM3) and single-nt mismatched sgRNA (M3-1), respectively. The x-axis represents the ratio of EGFP disruption percentage by M3-1 over PM3 sgRNA. Lower bars indicate higher fidelity. (TIF) [file pbio.3000747.s003.tif]

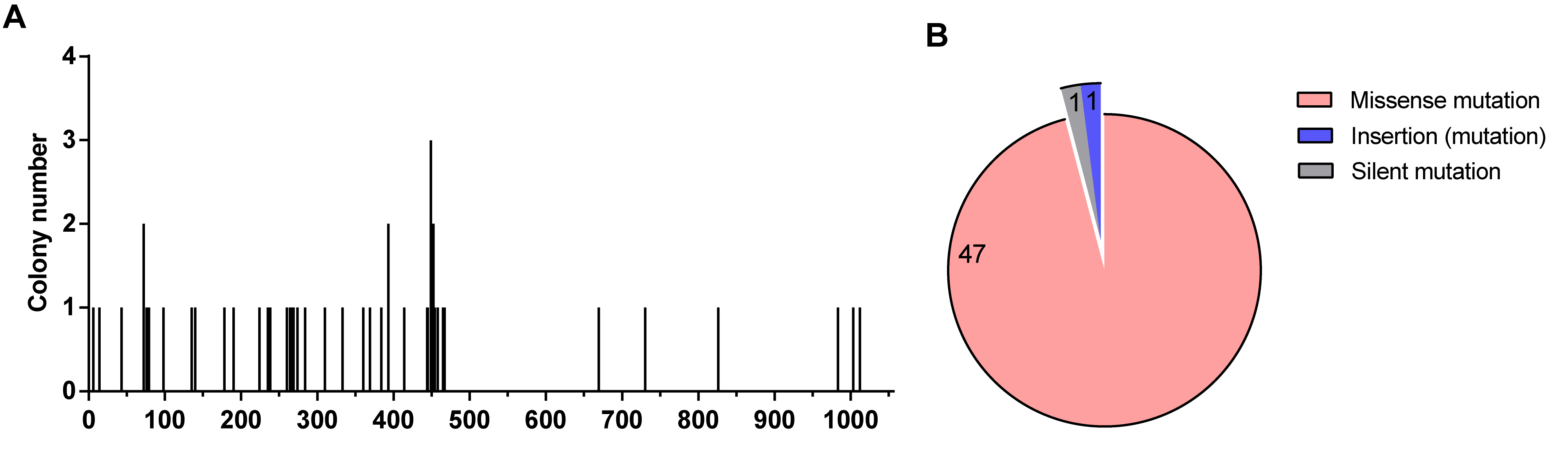

Supplement: S4 Fig — (A) Distribution and frequency of amino acid substitutions of 22 SaCas9-HF variants. Each mutation from the SaCas9 variants was mapped to the coding sequence of SaCas9. (B) Pie chart for the mutation type of 22 SaCas9-HF variants. (TIF) [file pbio.3000747.s004.tif]

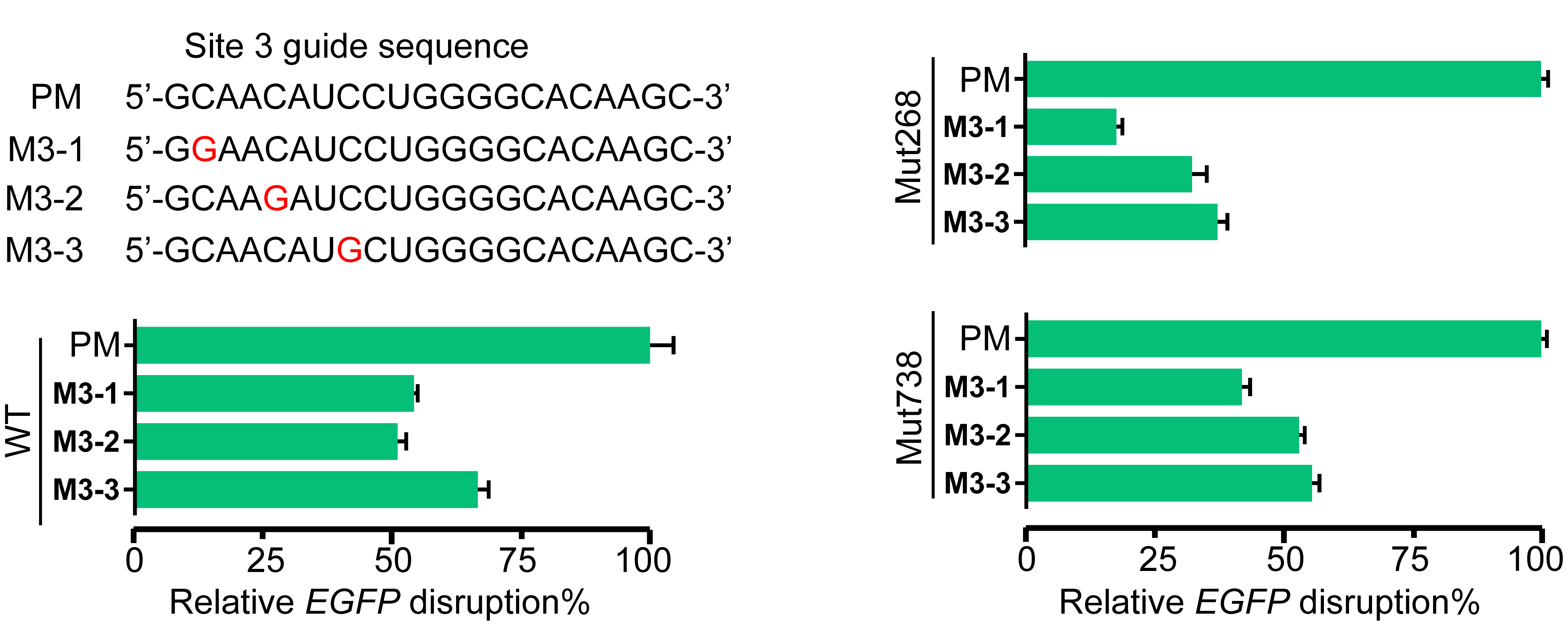

Supplement: S5 Fig — Two SaCas9 mutants’ activity and fidelity were investigated. (TIF) [file pbio.3000747.s005.tif]

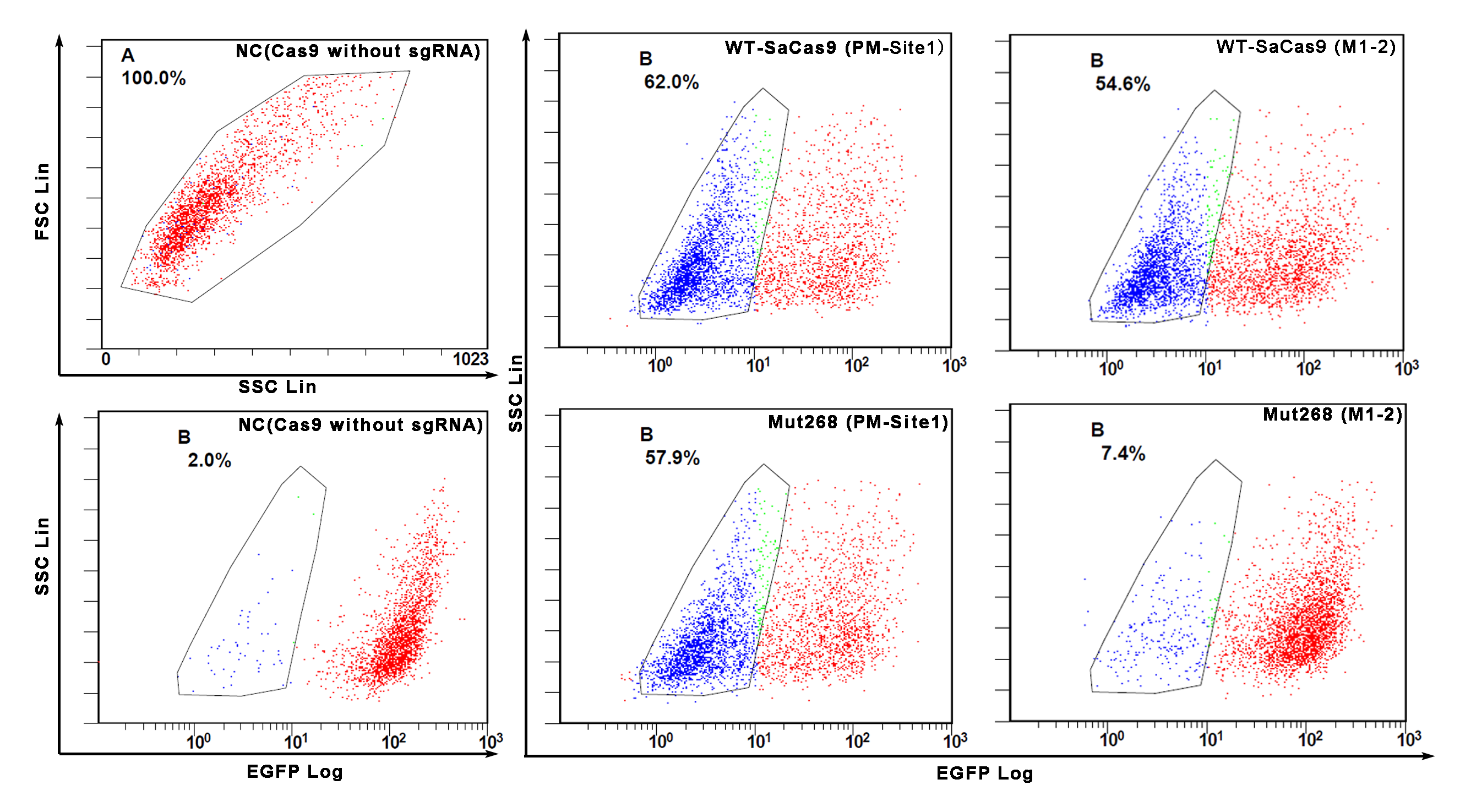

Supplement: S6 Fig — sgRNA was designed to target site 1, and mismatched sgRNA (M1-2) was used to test the fidelity of SaCas9. FCM results for Fig 1F. (TIF) [file pbio.3000747.s006.tif]

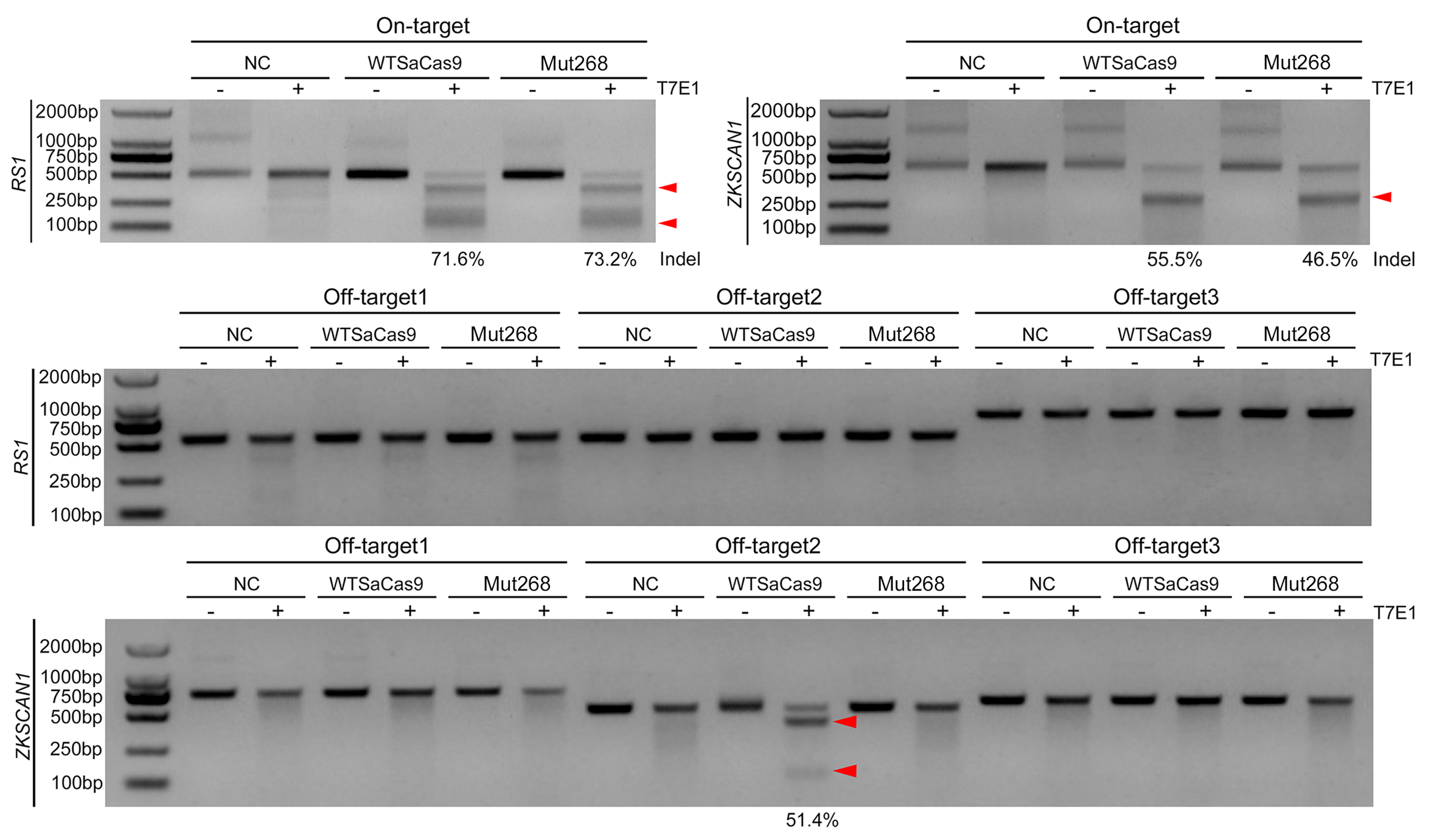

Supplement: S7 Fig — Red arrows represent cleaved bands. The percentage represents the cut efficiency. NC represents negative control. (TIF) [file pbio.3000747.s007.tif]

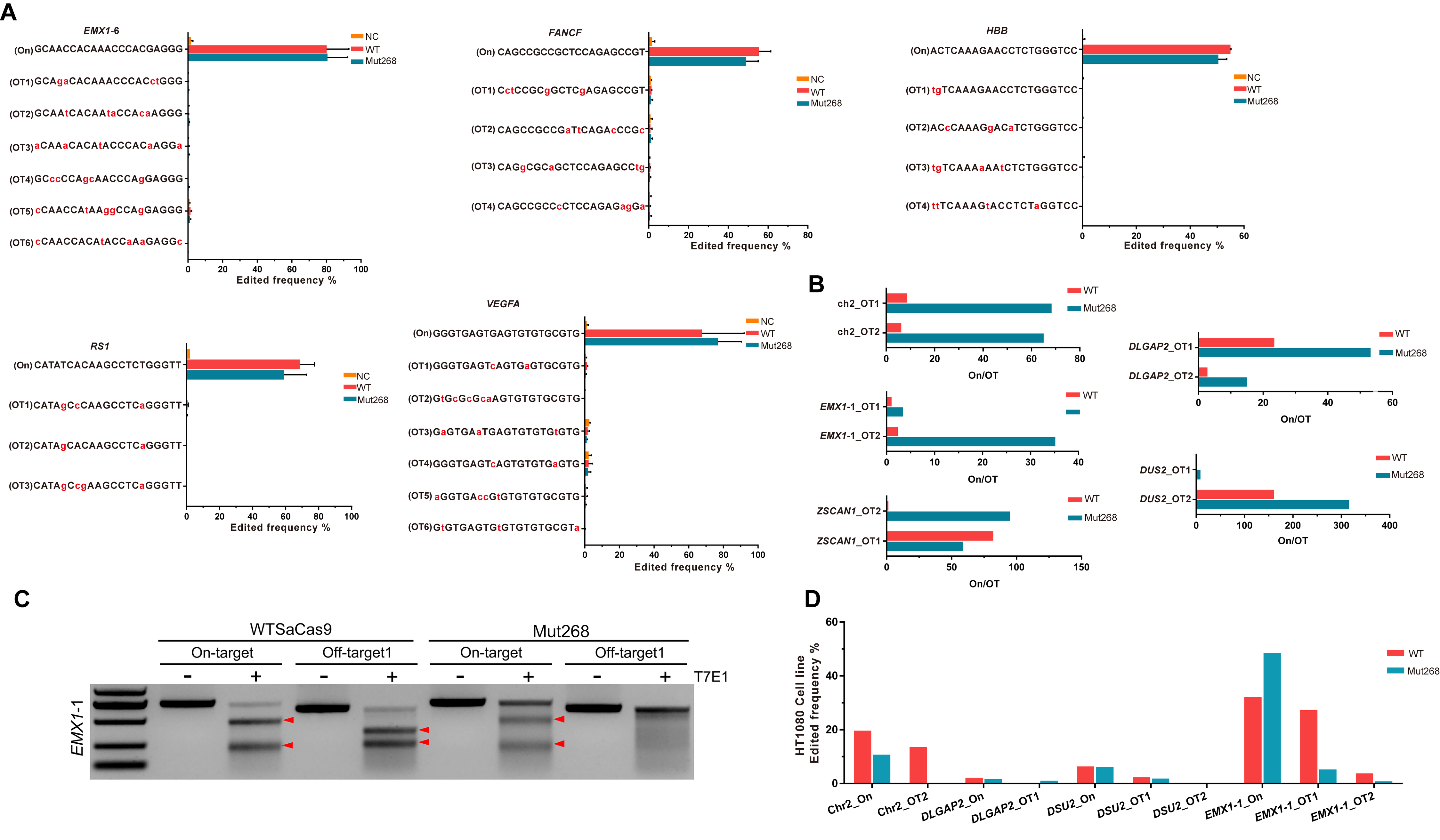

Supplement: S8 Fig — (A) WT and Mut268 mediated cleavage at on target (On) and predicted off target (OT) sites measured by targeted deep sequencing. (B) On-/off-target ratios were calculated from the data in Fig 2B. (C) T7EI assay for the specificity of Mut268 at EMX1-1 site. (D) WT and Mut268 mediated cleavage in HT-1080 cell lines. (TIF) [file pbio.3000747.s008.tif]

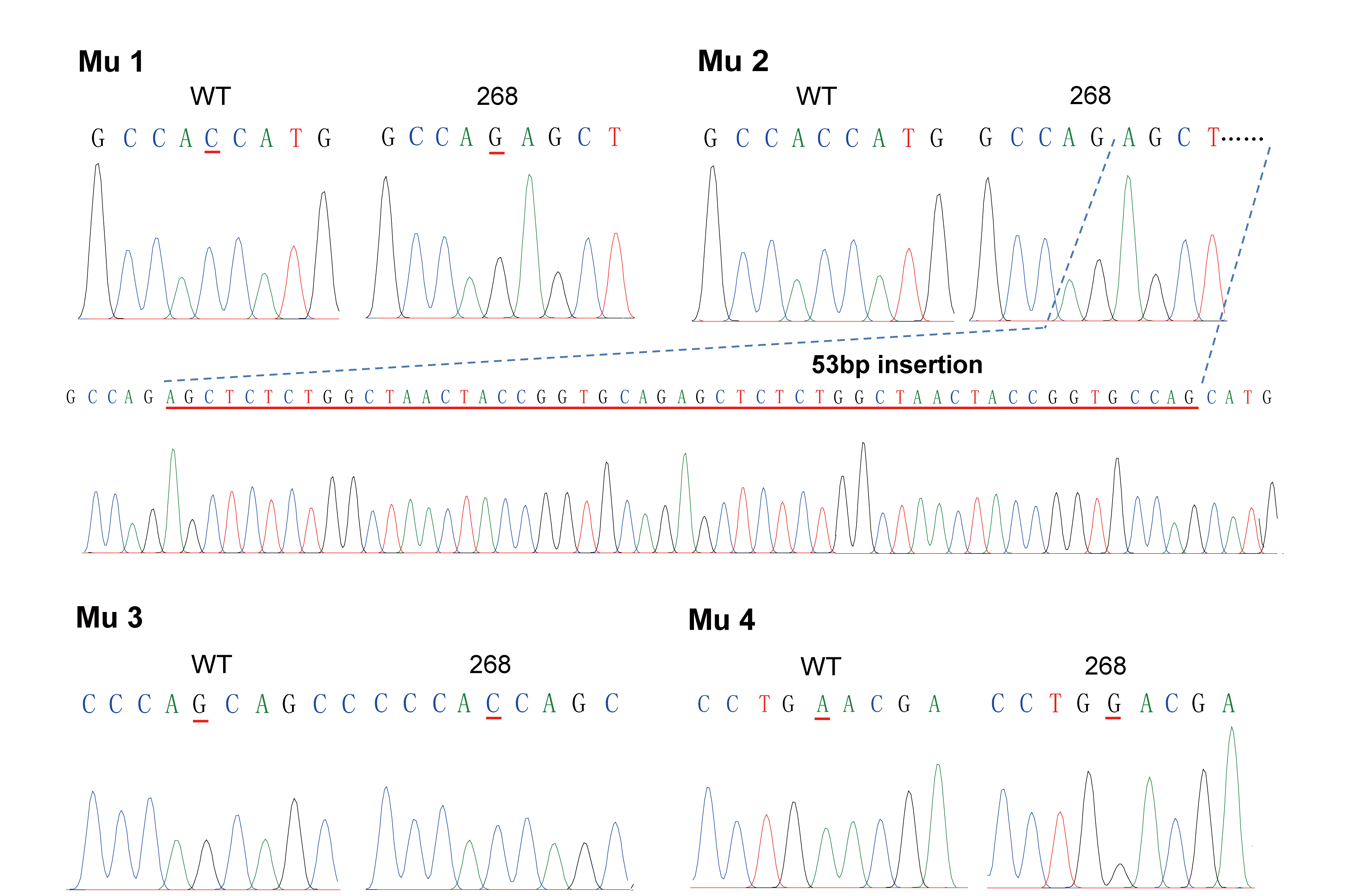

Supplement: S9 Fig — DNA sequencing analysis of Mut268 revealed 4 mutations (Mu1–Mu4). WT and 268 represent the WT SaCas9 and Mut268, respectively. (TIF) [file pbio.3000747.s009.tif]

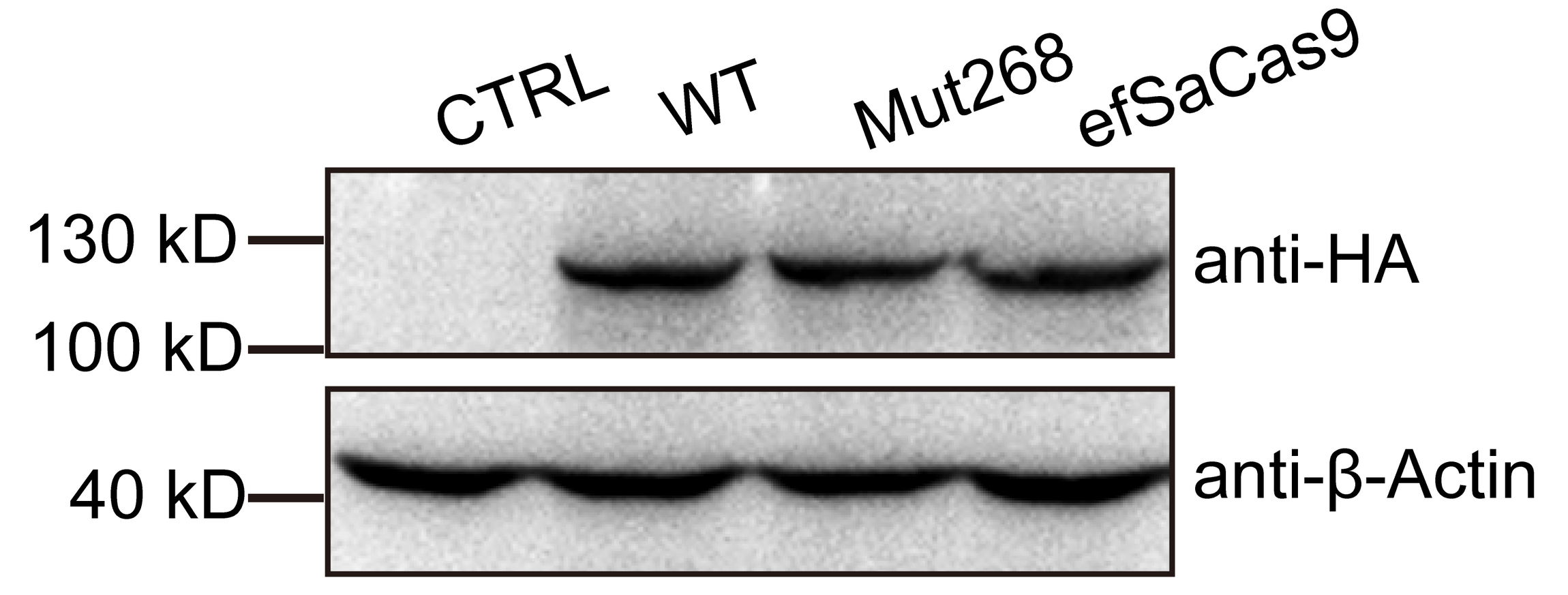

Supplement: S10 Fig — HA-tagged SaCas9 and its variants were analyzed by western blotting using the indicated antibodies. CTRL, HEK-293 cells without transfection; WT, wild-type SaCas9 (pX601); Mut268, Mut268 variant; efSaCas9, N260D variant. (TIF) [file pbio.3000747.s010.tif]

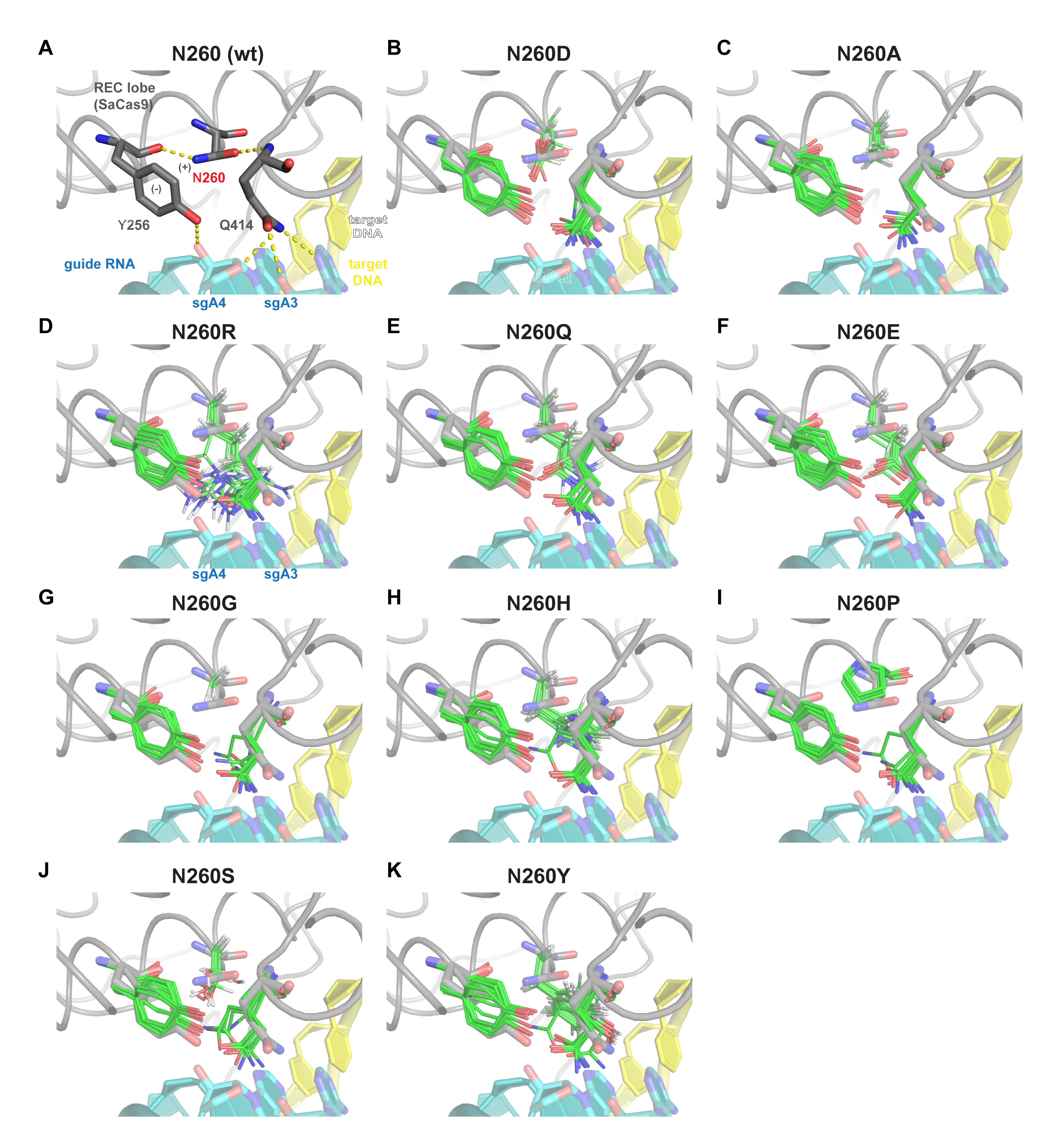

Supplement: S11 Fig — (A) Hydrogen bonding and cation-π interactions mediated by N260 in WT ScCas9. PDB ID:5CZZ. (B–K) Ten lowest-energy structures simulated of SaCas9 mutants by Rosetta Backrub: N260D (B), N260A (C), N260R (D), N260Q (E), N260E (F), N260G (G), N260H (H), N260S (J), and N260Y (K). Only the positions of the side chains of Y256, 260, and Q414 (green sticks) are shown for clarity. N260C simulation was not performed, as cysteine simulations are presently not available through Rosetta Backrub. (TIF) [file pbio.3000747.s011.tif]

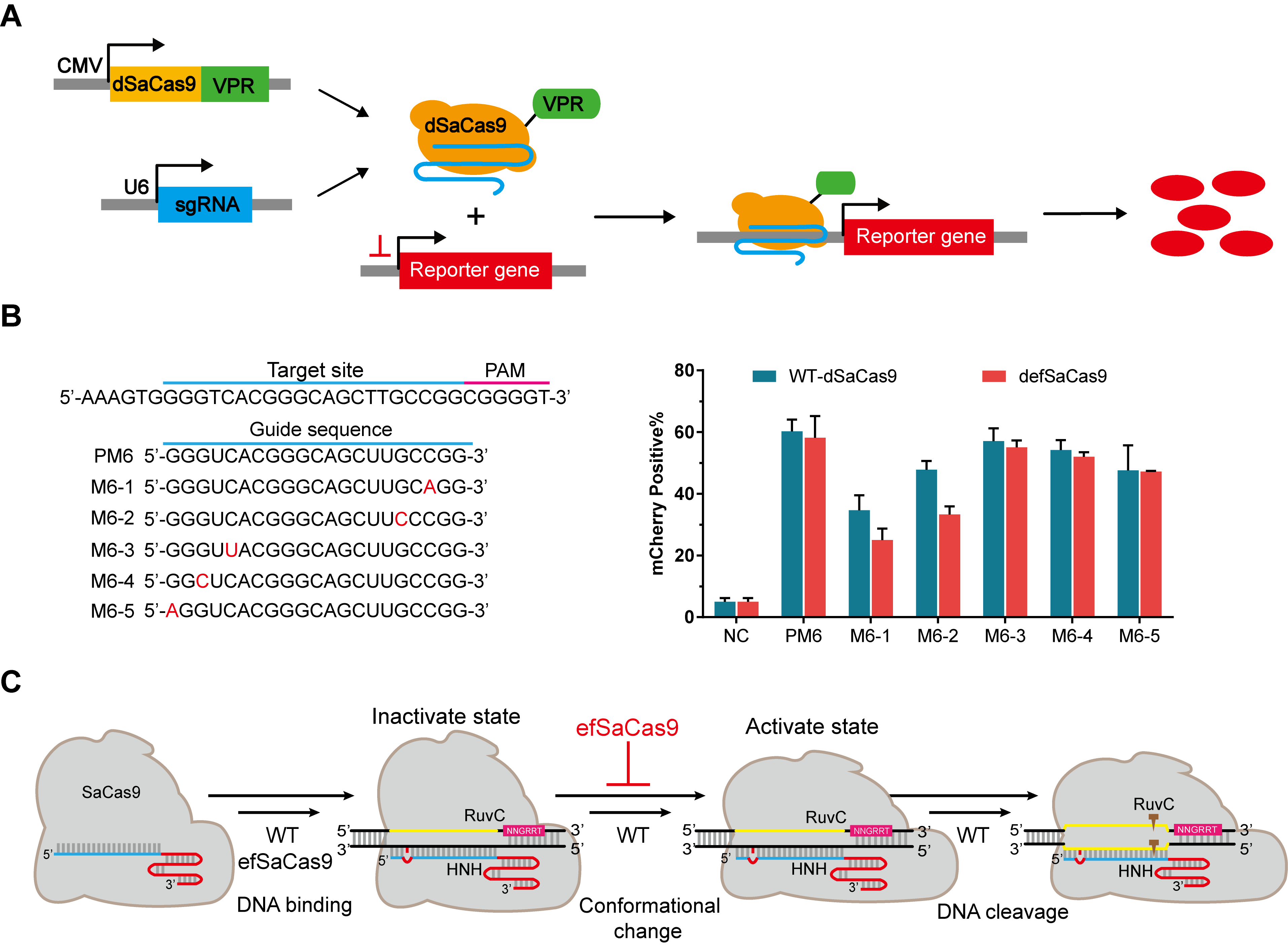

Supplement: S12 Fig — (A) Schematic representation of miniCMV-mCherry based transcriptional activator reporter. Upon binding of dSaCas9-VPR to miniCMV promoter, mCherry expression is activated. (B) mCherry activation was measured in HEK-293 cells transfected with dSaCas9-WT or defSaCas9 based transcriptional activators plus perfectly matched sgRNA or mismatched sgRNAs, as indicated; error bars, SEM; n = 3. (C) Model for enhanced fidelity of efSaCas9. Mutation in REC lobe may increases the threshold for HNH activation and cleavage when SaCas9 targets the mismatched RNA–DNA heteroduplex. (TIF) [file pbio.3000747.s012.tif]

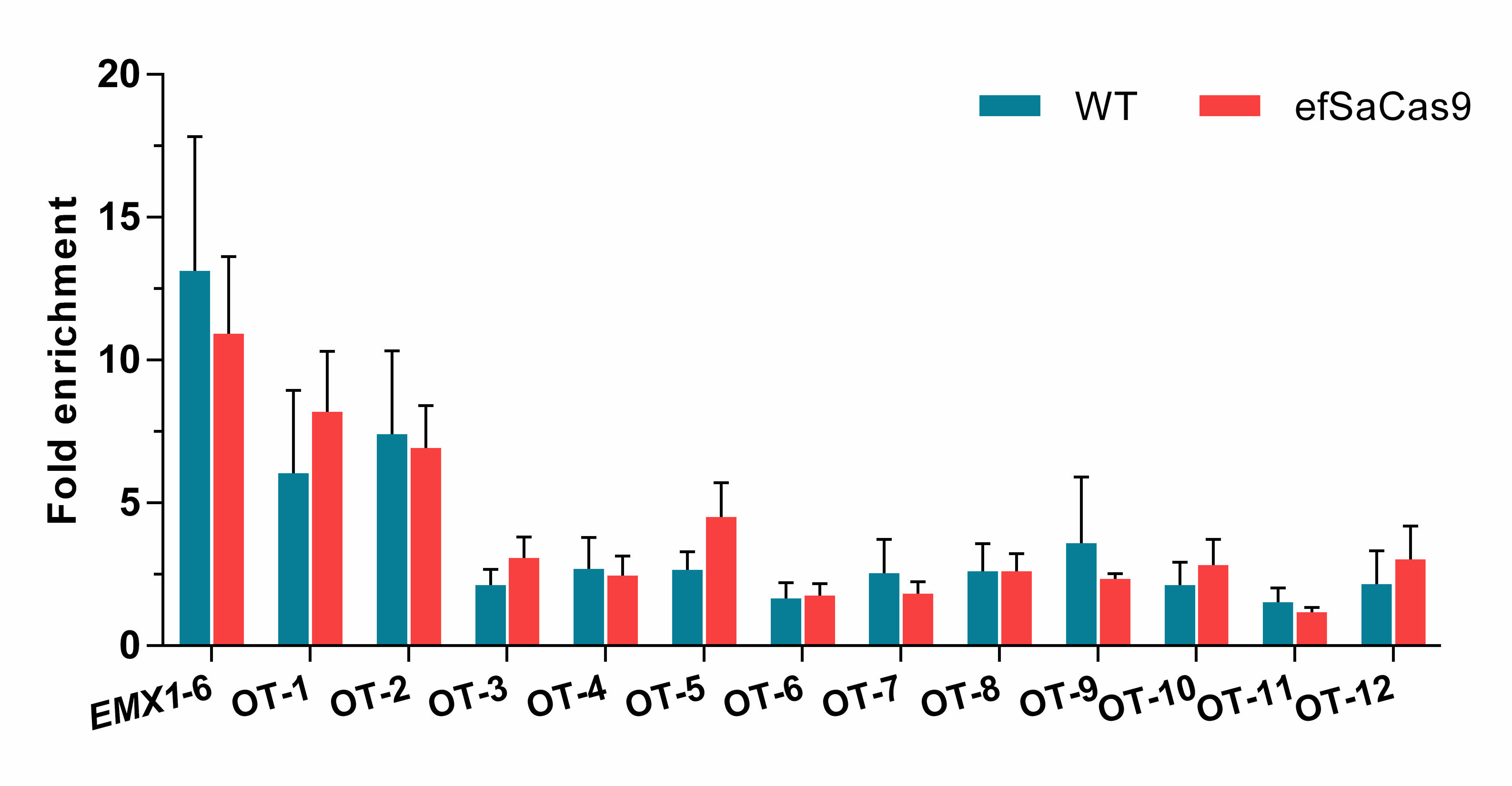

Supplement: S13 Fig — Primers for qPCR were in supplementary S2 Table. Off target (OT) sites were predicted and measured by qPCR. (TIF) [file pbio.3000747.s013.tif]

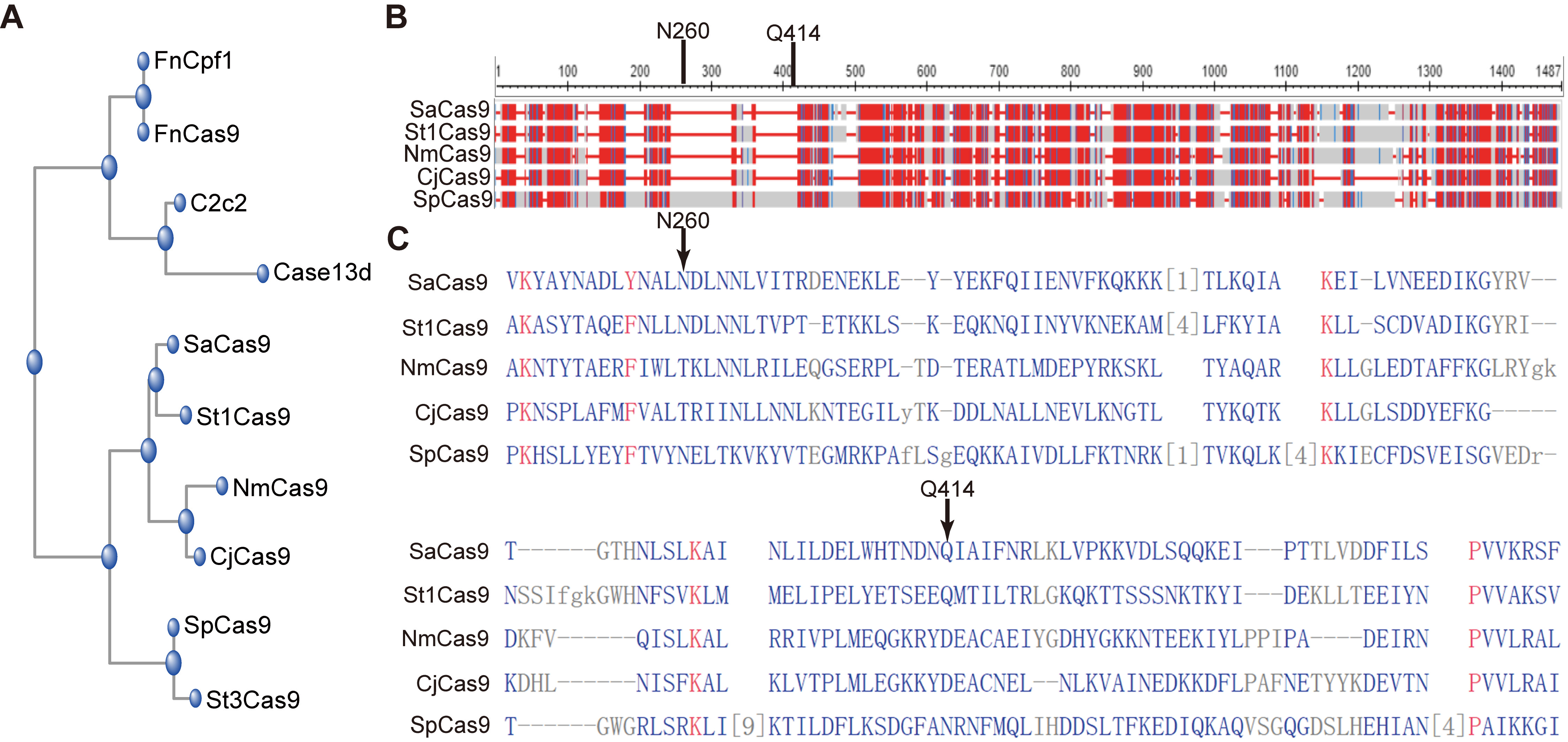

Supplement: S14 Fig — (A) Phylogenetic tree of Cas proteins. Phylogenetic tree was generated with COBALT software. (B) Alignment results of 5 Cas9 proteins. (C) Part of alignment results. Highly conserved sequences are shown in red. The N260 and Q414 residues of SaCas9 are marked by arrows. (TIF) [file pbio.3000747.s014.tif]

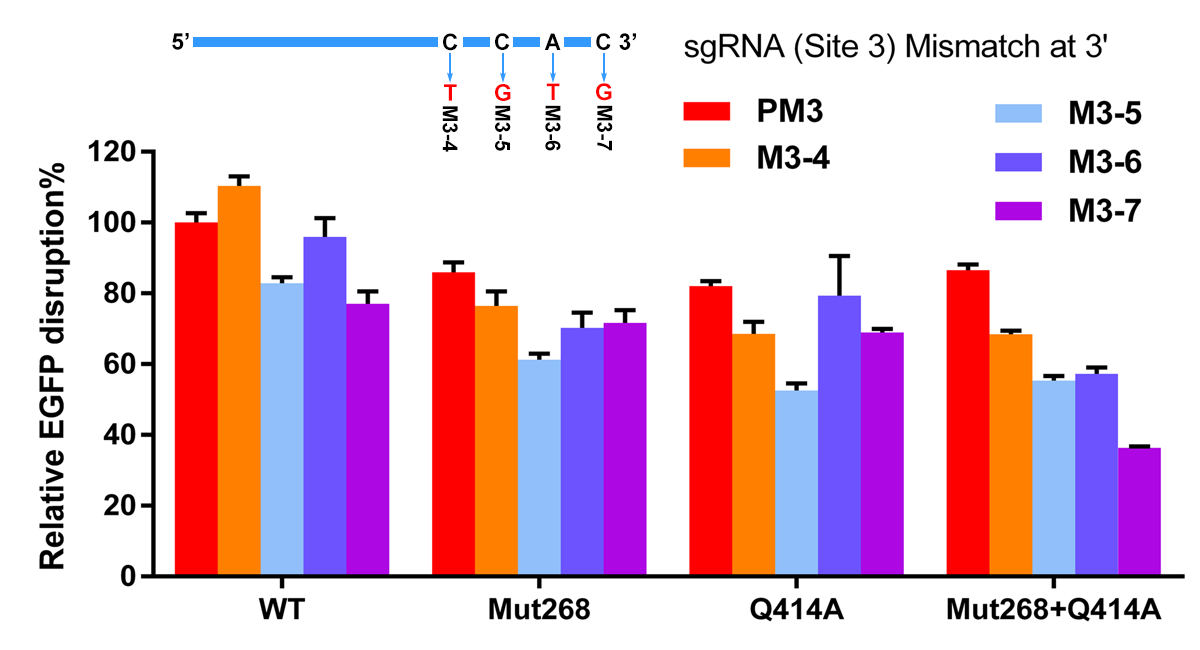

Supplement: S15 Fig — Fidelity comparisons of structure-guided additional SaCas9 mutants with perfect-matched sgRNA 3 (PM3) and corresponding single-nt mismatched sgRNAs (M); error bars, SEM; n = 3. Relative disruption efficiencies are normalized to perfect-matched sgRNA of WT SaCas9. (TIF) [file pbio.3000747.s015.tif]
